# 2266 Big data approaches in translational science: The influence of psychiatric and trauma history in predicting smoking during pregnancy in a cohort of female like-sex twin pairs

**DOI:** 10.1017/cts.2018.156

**Published:** 2018-11-21

**Authors:** Alexandra N. Houston-Ludlam, Julia D. Grant, Kathleen K. Bucholz, Pamela A. F. Madden, Andrew C. Heath

**Affiliations:** 1 Washington University in St. Louis; 2 Department of Psychiatry, Washington University in St. Louis

## Abstract

OBJECTIVES/SPECIFIC AIMS: Smoking during pregnancy (SDP) is associated with negative health outcomes, both proximal (e.g., preterm labor, cardiovascular changes, low birth weight) and distal (e.g., increased child externalizing behaviors and attention deficit/hyperactivity disorder (ADHD) symptoms, increased risk of child smoking). As pregnancy provides a unique, strong incentive to quit smoking, investigating SDP allows analysis of individual predictive factors of recalcitrant smoking behaviors. Utilizing a female twin-pair cohort provides a model system for characterizing genotype×environment interactions using statistical approaches. METHODS/STUDY POPULATION: Using women from the Missouri Adolescent Female Twin Study, parental report of twin ADHD inattentive and hyperactive symptoms at twin median age 15, and twin report of DSM-IV lifetime diagnosis of major depressive disorder, trauma exposure (physical assault and childhood sexual abuse), collected at median age 22, were merged with Missouri birth record data for enrolled twins, leading to 1553 individuals of European ancestry and 163 individuals of African-American ancestry included in final analyses. A SDP propensity score was calculated from sociodemographic variables (maternal age, marital status, educational attainment, first born child) and used as a 6-level ordinal covariate in subsequent logistic regressions. RESULTS/ANTICIPATED RESULTS: For European ancestry individuals, parental report of hyperactive ADHD symptoms and exposure to childhood sexual abuse were predictive of SDP, while a lifetime diagnosis of major depressive disorder, parental report of inattentive ADHD symptoms, and exposure to assaultive trauma were all not significantly predictive of future SDP. For African-American individuals, none of these variables were significant in predicting future SDP. DISCUSSION/SIGNIFICANCE OF IMPACT: Understanding this relationship of risk-mechanisms is important for clinical understanding of early predictors of SDP and tailoring interventions to at risk individuals. Ultimately, the focus of this research is to mitigate risk to pregnant smokers and their children. Additionally, the cohort-ecological approach informs how well research and administrative (vital record) data agree. This allows for evaluation of whether administrative data improve prediction in research cohorts, and conversely if research data improve prediction over standard sociodemographic variables available in administrative data.

